# The Fetal Alcohol Spectrum Disorders—An Overview of Experimental Models, Therapeutic Strategies, and Future Research Directions

**DOI:** 10.3390/children11050531

**Published:** 2024-04-28

**Authors:** Magdalena Król, Paweł Skowron, Kamil Skowron, Krzysztof Gil

**Affiliations:** 1 Department of Pathophysiology, Jagiellonian University Medical College, Czysta St. 18, 31-121 Krakow, Poland; magdalena.krol@doctoral.uj.edu.pl (M.K.); kamil.skowron@uj.edu.pl (K.S.); 2 Department of Physiology and Pathophysiology, Wroclaw Medical University, T. Chalubinskiego St. 10, 50-368 Wrocław, Poland; pawel.skowron@student.umw.edu.pl

**Keywords:** fetal alcohol spectrum disorder, fetal alcohol spectrum, prenatal alcohol exposure, experimental models, interventions, pregnancy, alcohol, fetal

## Abstract

Since the establishment of a clear link between maternal alcohol consumption during pregnancy and certain birth defects, the research into the treatment of FASD has become increasingly sophisticated. The field has begun to explore the possibility of intervening at different levels, and animal studies have provided valuable insights into the pathophysiology of the disease, forming the basis for implementing potential therapies with increasingly precise mechanisms. The recent reports suggest that compounds that reduce the severity of neurodevelopmental deficits, including glial cell function and myelination, and/or target oxidative stress and inflammation may be effective in treating FASD. Our goal in writing this article was to analyze and synthesize current experimental therapeutic interventions for FASD, elucidating their potential mechanisms of action, translational relevance, and implications for clinical application. This review exclusively focuses on animal models and the interventions used in these models to outline the current direction of research. We conclude that given the complexity of the underlying mechanisms, a multifactorial approach combining nutritional supplementation, pharmacotherapy, and behavioral techniques tailored to the stage and severity of the disease may be a promising avenue for further research in humans.

## 1. Introduction

The establishment of a safe threshold for prenatal alcohol exposure (PAE) remains elusive, as current international guidelines strongly discourage the consumption of any quantity or form of alcohol during pregnancy. However, it is worth noting that approximately 10% of pregnant women across the globe engage in alcohol use [1,2]. Fetal alcohol spectrum disorders (FASDs) are prevalent across many socioeconomic and ethnic backgrounds, indicating their widespread occurrence. Every day, a staggering number of over 1700 new cases of fetal alcohol spectrum disorder (FASD) are diagnosed worldwide. This alarming statistic highlights the significant impact on public health and the global economy, as well as the misery experienced by affected individuals and their families [3].

Since Jacqueline Rouquette (1957) [4] and Paul Lemoine (1968) [5] established a clear link between maternal alcohol consumption during pregnancy and certain birth defects, which was later popularized by Kenneth Lyons Jones and David W. Smith (1973) [6], the research into the treatment of FASD has become increasingly sophisticated. However, the current research emphasizes the need to improve our understanding of the multifactorial pathophysiology of the disease, which will serve as the basis for future experimental studies aimed at identifying novel therapeutic interventions [7]. A number of mechanisms have been proposed to be central to the development of FASD, including oxidative stress, disruption of developmental signaling and migration pathways, and neural cell death. Alcohol metabolism causes oxidative damage that leads to neural crest cell apoptosis and defects in the signaling processes responsible for morphogenesis and organogenesis. Alcohol-induced activation of glial cells, with production of pro-inflammatory mediators and reactive oxygen species, promotes neuroinflammation. Moreover, alcohol has been demonstrated to interfere with the activity of key morphogens and growth factors, which contributes to craniofacial defects. The devastating effects in the central nervous system are already evident at the level of epigenetic regulation of gene expression. It has also been shown to affect the plasticity of synapses, which depends on the function of glutamatergic and GABAergic neurons. Additionally, the hypothalamic–pituitary–adrenal (HPA) axis and the microbiota–gut–brain axis are both affected, with varying degrees of severity. These complex mechanisms appear to be the major contributors to the development of the heterogeneous clinical presentation of the disease, but their detailed description is beyond the scope of this paper and has been extensively discussed in other publications [7,8].

The objective of this article is to provide a comprehensive overview of the latest research and reports on experimental research on FASD. The article commences with an overview of experimental animal models of FASD and then subsequently examines the interventions utilized in these models, highlighting any potentially beneficial therapeutic effects.

## 2. Materials and Methods

**Figure 1:**
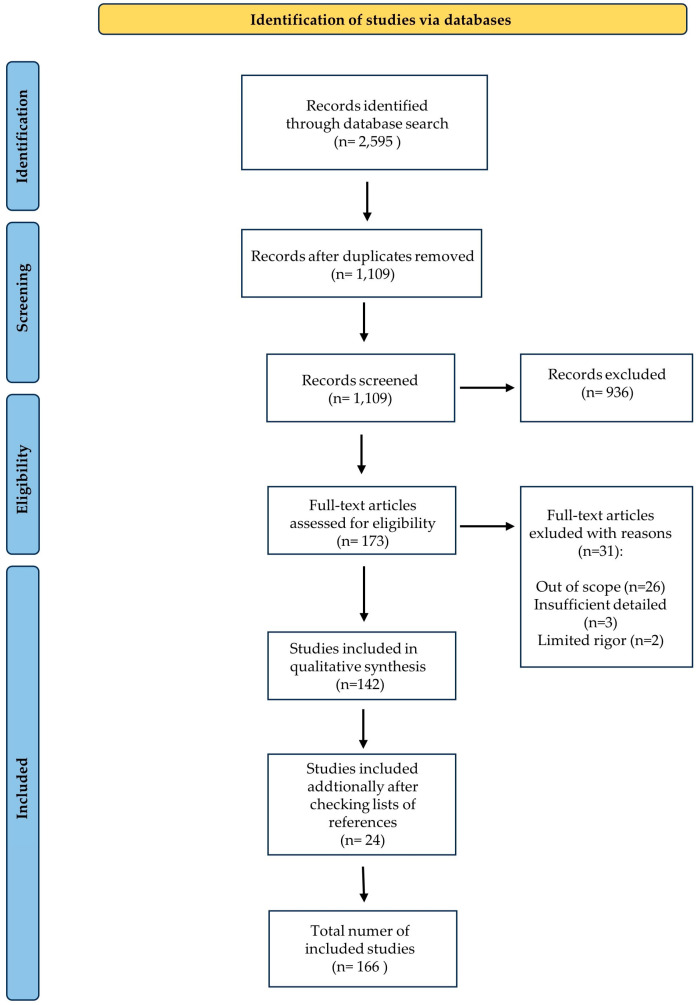
The flow chart of the publication selection process.

This paper is in narrative form and is based on both original and review articles written in English and indexed in Medline, Scopus, Web of Science, and Google Scholar databases, selected for their relevance in describing the chosen scientific topic. These databases were searched up to November 2023 with the use of the following terms: ‘fetal alcohol spectrum disorders’ and ‘animal models’ or ‘dietary supplements’ or ‘vitamin supplementation’ or ‘postnatal intervention’ or ‘diet therapy’ or ‘drug therapy’ or ‘prevention and control’ or ‘rehabilitation’ or ‘postnatal supplementation’ or ‘therapy’ or ‘postnatal care’ or ‘*Drosophila melanogaster*’. The evaluation of titles and abstracts was carried out by two reviewers independently, with a focus on the key research related to the main topic. Any disagreements in assessing the articles were discussed to reach unanimous consensus. We decided to include both original studies and review articles. Moreover, we checked the lists of references of the included papers for additional relevant articles. Full-text articles were obtained on the basis of their significance to the study. The flow chart of the publication selection process is shown in Figure 1.

## 3. Types of Experimental Models of FASD

Experimental models have been widely and effectively adopted for the investigation of human diseases [9]. They have the potential to surmount obstacles that impede research in the clinical population and also offer a genetically manipulable framework that facilitates reliable and replicable analysis. Additionally, they offer a level of control over genetic and environmental factors that would otherwise be unattainable [10,11]. Below, we have included a brief description of the different types of experimental models used to study fetal alcohol syndrome, and their key features are summarized in Table 1.

### 3.1. Zebrafish Models

The zebrafish emerged as a prominent model system during the early 1980s, as a result of the efforts of George Streisinger and Charles Kimmel and a group of researchers [11,12]. Over 70% of genes exhibit homology between the genomes of humans and zebrafish, and this percentage increases to 82% when the genes are identified as the causative factors of human diseases [13]. The zebrafish exhibits a range of experimental, biological, and behavioral traits that render it an appropriate model organism for investigating the effects of embryonic ethanol exposure on development [11]. The initial utilization of zebrafish in the field of FASD mostly centered on the examination of ethanol-induced anatomical abnormalities. Blader and Strähle, in 1998, conducted a study to investigate the similarities between holoprosencephaly and certain children with FASD. They found that administering 2.4% ethanol within a specific time frame, from the dome stage to 30% epiboly, resulted in the development of cyclopic phenotypes that resembled those observed in wnt11 mutants, where the Wnt/PCP signaling pathway is impaired [9,14]. The foregoing findings provide evidence that ethanol has an impact on the motions of cells during gastrulation. The Wnt/PCP pathway plays a crucial role in the process of gastrulation movements and exhibits a genetic interaction with a 1% ethanol concentration, resulting in the manifestation of cyclopia in *vangl2* mutants. These mutants also have disturbances in Wnt/PCP signaling [15].

Thanks to the small size of Zebrafish embryos, a significant number of subjects can be accommodated conveniently and cost-effectively. In addition, the rapid embryonic development and abbreviated time to achieve sexual maturity enable efficient breeding and cross-generational investigations. However, of utmost significance in the context of FASD research, this method provides a high level of accuracy and comprehensive regulation in terms of the timing, dosage, and duration of alcohol exposure to the developing embryo. This is due to the fact that the eggs are fertilized and undergo development outside the maternal organism [16].

To terminate the exposure to ethanol, the researchers extract embryos from the ethanol solution and transfer them to an untreated medium. Therefore, the utilization of zebrafish provides a convenient means to investigate acute, distinct, or prolonged ethanol exposures throughout a broad spectrum of doses in many embryos concurrently [11].

In 2021, Schaidhauer et al. summarized that establishing a direct comparison between zebrafish and human development is challenging. However, they attempted to establish a connection between zebrafish development and the second and third trimesters of human development. This particular period is characterized by significant cell migration and differentiation, as well as the initiation of brain growth, pruning, and refinement. These developmental processes in zebrafish occur until approximately 120 h post-fertilization [3,17].

### 3.2. Rodent Models

#### 3.2.1. Mouse Models

Mouse models are one of the most commonly used PAE animal models. They have several advantages that are crucial to researchers. Mice are easy to care for, they have many biological and physiological similarities to humans, and they are the most genetically similar animal model. Genetic engineering tools are available to genetically modify mice, allowing researchers to study the contribution of specific genes, genetic variants, or signaling pathways. The teratogenic effect of alcohol exposure, including craniofacial and brain malformations, has been well described in mouse models, giving researchers a basis for a comparison of their own data [18,19]. The main disadvantage of the mouse model is that the equivalent of the third trimester is considered to occur after birth from days 1–10 postnatally. This implies differences in alcohol absorption, distribution, metabolism, and elimination compared to humans in utero due to the lack of a placental barrier.

The timing, dosage, and route of alcohol administration play a crucial role in the study outcome. There are few recognizable patterns of alcohol exposure and route of administration. Intraperitoneal injection and intragastric gavage result in high blood alcohol concentration (BAC), so they are preferable in models of acute exposure. Their disadvantage is that they are stressful for the animals. Voluntary ethanol feeding results in low to moderate BAC, so it is preferable in chronic exposure models. This route prevents stress caused by other invasive methods, but on the other hand, it is difficult to control the dosage and time of alcohol administration [20]. As the equivalent of the third trimester occurs in mice postnatally, it implicates different approaches in alcohol administration. Alcohol exposure can come through the mother feeding her pups or directly delivering to pups. It should be noted that most mouse strains will not drink alcohol voluntarily in a significant amount, except for C57BL/6J, which has a high preference for alcohol [21].

The harmful effects of alcohol depend on the stage of fetal development in which exposure to alcohol occurred. Mouse gestation days 1–10 are equivalent to the first trimester in humans. When this animal is exposed to alcohol during the period corresponding to the first trimester of human, we can observe mainly craniofacial dysmorphologies and also brain malformations and modified grey and white matter tracts [22,23,24]. In mice, the period corresponding with the second trimester occurs on 11–21 days of gestation. The exposure to alcohol in this period may result in malformations of craniofacial and brain, neurodevelopmental disorders, or restriction of fetal growth [23,25,26,27,28,29]. The third trimester in mice is considered to be the 10-day postnatal period after birth and the harmful effects of alcohol during that time include brain abnormalities and neurodevelopmental disorders [30,31,32,33]. Interestingly, the work of Petrelli et al. suggests that serious craniofacial malformations usually present with the use of the acute PAE model during the first trimester. On the other hand, the results of impaired behaviors, remembering both deficits in learning and behaviors resembling symptoms of anxiety or depression, are best studied using the chronic PAE model of the third trimester [34].

#### 3.2.2. Rat Models

Another example of a rodent model used in research on FASD is a rat model. An evident benefit of rats is their greater size, facilitating handling and sampling processes. Additionally, due to the increased complexity of behaviors, for example, learning and memory tests or executive function assessments, they are a more suitable choice for experimentation concerning the behavioral aspects of PAE [35].

Similarly to a mouse model, the rat model provides an opportunity for various methods of ethanol administration. First, chronic exposure, which involves continuous exposure throughout gestation, can be achieved through liquid diet or voluntary drinking paradigms [36,37,38]. Alternatively, when we desire BACs, we can apply oral intubation [39]. This method can be employed during the entire gestation period, only during the third trimester equivalent [40,41], or throughout the equivalents of all three [42,43]. Nowadays, we infrequently included vapor inhalation in the protocols, whereas injection of ethanol intraperitoneally or subcutaneously is not usually performed in rat models [44,45]. This method is typically reserved for mouse models, where the impact of ethanol on neuroanatomical aspects is investigated [19,46].

Furthermore, rats have exhibited all the characteristic traits of FASD, such as growth retardation, physical deformities, damaged neurological function, and cognitive impairments [40,42,47,48,49,50]. Interestingly, in 1989, Abel designed a study to verify whether effects of paternal alcohol consumption differ from the effects of maternal alcohol consumption. The presented results indicated that paternal alcohol consumption led to a decrease in litter size in offspring, a dose-dependent decrease in testosterone levels, a decrease in testosterone/estradiol ratio, or changes in offspring activity. However, this pattern of alcohol consumption was not linked to lower birth weight, increased postnatal mortality, or poorer passive avoidance learning, while in the case of maternal alcohol consumption, these findings were observed [50]. 

In 2005, Christie et al. conducted a study on rats that presented that prenatal exposure to ethanol can result in long-lasting impairments in both reference and working memory that persist into adulthood. These deficiencies are accompanied by a diminished capability of the hippocampus dentate gyrus to sustain long-lasting long-term potentiation (LTP) [51]. Similarly, in 2002, Nixon et al. discovered that binge ethanol exposure reduced the number of bromodeoxyuridine-positive cells, indicating a decrease in neural progenitor cell proliferation in the adult dentate gyrus [52].

These and other studies show that the research on fetal alcohol syndrome in a rat model provides a wealth of important information on the pathogenesis and potential mechanisms of damage induced during PAE. Furthermore, it is feasible to evaluate potential therapeutic interventions using rat models of FASD. Multiple therapies administered either simultaneously with ethanol or after ethanol exposure (such as providing supplements to offspring after birth) exhibit potential for alleviating or reversing certain cognitive impairments linked to FASD [53,54].

### 3.3. Xenopus Model

Amphibian models play a key role in early development research. Since the 1950s, Xenopus has been widely used to better understand the mechanisms of embryogenesis and organogenesis [55]. Frogs are oviparous organisms that lay a large number of eggs that can be easily fertilized in vitro and, therefore, can produce a large number of embryos in a few days during all seasons [56]. Due to the relatively large size of the xenopus embryos, they can be easily manipulated. Moreover, because of the fact that embryonic development takes place outside the mother’s body, it can be studied throughout all stages of development. Another advantage of the Xenopus model is that they are cultured in aqueous conditions, allowing the simple addition of ethanol. One of the reasons for the significant role of Xenopus in the research of various human diseases is its similarity to higher vertebrates in terms of organ development, physiology, and gene expression [57]. The advantages mentioned above led to the development of The Frog Embryo Teratogenensis Assay-Xenopus (FETAX), which is used to study the teratogenic effect of different pollutants in this animal model’s early stages of development. FETAX is also used to test mortality and malformations after exposure to ethanol [58]. With the Xenopus model, researchers are also able to measure simple behaviors to assess the effects of alcohol exposure on cognitive functions [59,60].

The main drawback of the frogs as an ethanol exposure model is that they are oviparous organisms. Thus, the embryos develop externally to the mother’s body, in contrast to humans, who are placental mammals, and the development of the embryo occurs in connection with the mother. This implicates differences in prenatal exposure to alcohol, which, in humans, comes after absorption, distribution, and metabolism through the mother and not direct exposure as it comes in the oviparous model. Another disadvantage of the Xenopus model is the long generation time of about 1 year. This long period of development limits the use of adult animals raised from alcohol-exposed or experimentally modified embryos [60].

Nevertheless, studies have shown that exposure to ethanol in Xenopus causes outcomes similar to those in humans, such as neurological disorders, growth retardation, and deficiencies in the central nervous system. Due to the Xenopus model, we have a better understanding of molecular and biochemical understating of the etiology and pathophysiology of FASD, which may be helpful in identifying molecular targets assigned to possible strategies of therapy [61].

### 3.4. Avian Embryo Models

The avian embryo is probably the initial model organism in which the teratogenic effects of alcohol were examined [62].

Numerous studies conducted independently by Fere and Stockard indicated that exposure to ethanol through a vapor chamber resulted in elevated mortality rates, decreased development, and induced embryological abnormalities [62,63,64]. In 1968, Sandor and Elias conducted the initial assessment of alcohol’s developmental harm, specifically focusing on exposure during the gastrulation stage. This exposure, which accounted for 64% of the time spent in the air sac, hindered crucial processes in early morphogenesis. These included the disruption of the asymmetric blastoderm, the formation of smaller somites, decreased cranial flexure, reduced body length, and the occurrence of abnormalities in the brain, heart, otic vesicle, and caudal region [62].

One of the main advantages in using chick embryos for fetal alcohol syndrome research is the fact that local hatcheries offer fertile eggs at a reasonable price, making them appropriate for a wide range of research purposes [62]. The egg’s inherent confinement and consistent size enable strict regulation of dosage and exposure [65]. Additional benefits of the model encompass its simplicity in maintenance, minimal initial investment, and a non-burdensome Animal Care and Use Committee (ACUC) need when exclusively dealing with embryos [65,66].

Moreover, the application of the avian embryo model in the experimental studies allows for a variety of routes of exposure to ethanol. One of the methods is an in-ovo exposure that most accurately replicates natural development and causes the least amount of stress. Flentke et al. expanded a technique focused on the injection of alcohol diluted in isotonic saline. This is carried out using a precise needle and a glass microliter syringe into the yolk center of a horizontally positioned egg through a small hole made in the blunt end and air sac. It produces exposure kinetics that are very predictable, with the highest level of exposure occurring between 10 and 90 min after injection. There are similar kinetics in administration through air-sac injection, usually directly targeting the embryo [62]. Other methods include such routes as vapor exposure or shell-less incubation [67,68].

However, it is worth mentioning that applying alcohol directly to the embryo into a transparent egg should be avoided due to the osmotic effects that can cause indiscriminate harm. Regarding dosage, it is important that alcohol levels in the embryo are pharmacologically significant and do not exceed approximately 250 mg% (equivalent to 0.25% or 54 mmol/L) [62].

Nevertheless, the primary drawback of the chick embryo model is the unavailability of documenting the maternal factors that are associated with FASD. Contributions to this phenomenon involve various aspects such as the metabolic processing of alcohol and acetaldehyde by the mother’s organism, dietary influences, alterations in metabolic flow, disruptions in placental regulation, and the circulation between mother and fetus. On the other hand, it provides the opportunity to separate and study the specific impacts of alcohol on the embryo [62].

### 3.5. Caenorhabditis Elegans

One of the most commonly used invertebrate models of alcohol exposure is a fast-developing model of the nematode *Caenorhabditis elegans*. Due to its uncomplicated structure, it has some advantages over other animal models. The genome of *Caenorhabditis elegans* was fully sequenced and mapped, and its small nervous system consists of 302 neurons where each neuron has been described and its connectivity has been mapped. *C. elegans* has a rapid rate of development. During the first 36 h after being hatched, it goes through four post-embryonic stages of the larva, and just 68 h after being laid as an egg, it becomes mature enough to reproduce [69]. The main disadvantage of the *C. elegans* model is that its development occurs outside the body without the equivalent of a human placental barrier. Additionally, compared to simple vertebrate models such as Zebrafish or Xenopus, there is no assay to check the simple behaviors of *C. elegans*, so there is no possibility to assess the functional deficits of exposure to ethanol. Finally, the way alcohol is metabolized substantially differs from that in humans and it is hard to measure exact alcohol concentrations in the embryos [70].

Several studies have found that *C. elegans* is a feasible model for investigating the effects of ethanol exposure on the development both of physical skills and the nervous system. According to these reports, the embryo’s exposure to alcohol resulted in developmental delay, growth retardation, and reduced reproductive fecundity [69,70].

### 3.6. Drosophila Models

The *Drosophila melanogaster*, fruit fly, is a valuable research model for investigating the effects of developmental ethanol exposure. The rapid life cycle and heightened vulnerability to the teratogenic impact of alcohol on development have positioned Drosophila as a promising model for exploring the consequences of alcohol exposure [71]. Under controlled environmental conditions, it is possible to quickly and economically rear many individuals with the same genotype [72]. Flies in both their larval and adult stages can be exposed to alcohol either through ethanol vapor or by consuming food containing ethanol. However, due to the impermeable nature of the embryonic eggshell of Drosophila, special techniques are required to ensure embryonic ethanol exposure [73].

Interestingly, the teratogenic impact of alcohol on Drosophila exhibits parallels with FASD in humans. The research has demonstrated that when fed a diet containing ethanol, fruit flies experience developmental delays, display smaller larval central nervous systems, and exhibit a decrease in adult mass [74], as well as the disruption of neuronal development [73] and reduced viability [75]. Apart from the physical changes, we can also notice behavioral changes that are similar to those observed in mammal models. These include reduced food intake at all stages of development [76], hindered sensory processing [73], and disrupted sleep and activity patterns [75].

The advantage of this model is its simple genome, which enables a straightforward study of genetic factors that influence ethanol toxicity and altered gene expression after developmental ethanol exposure [72,74,75]. The access to genetic analysis makes Drosophila a great model for identifying developmental targets of alcohol. Logan-Garbisch et al. have explored the role of oxidative stress in developmental changes caused by alcohol and identified multiple mechanisms for ethanol-induced oxidative stress [77].

One of the limitations of the Drosophila model is the complexity in interpreting findings and contrasting them with mammalian models. An example of this is evaluating possible cognitive deficiencies that may arise following alcohol exposure during development, which can be quite challenging to assess [75].

## 4. Applications of the Experimental Model of FASD

**Figure 2:**
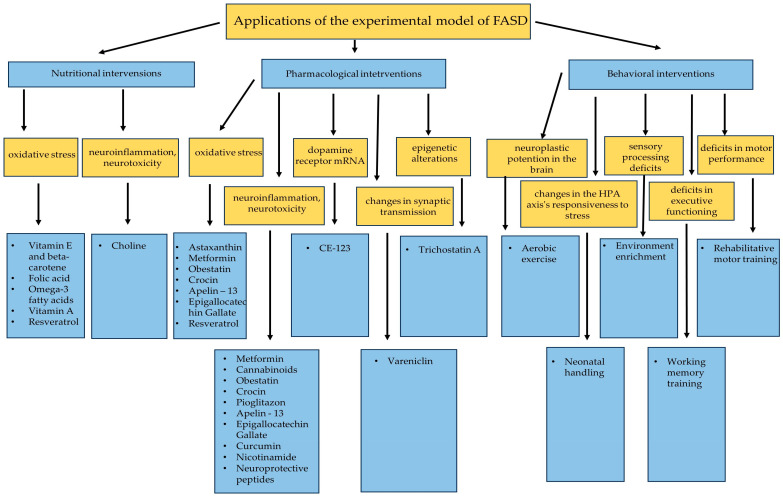
An overview of pathophysiological mechanisms targeted by interventions applied in experimental model of FASD.

Below, there are described the main interventions, including the nutritional, pharmacological, and behavioral approaches that were studied using the experimental animal models of FASD. The mechanisms responsible for producing harm in FASD have not been completely understood yet. Nevertheless, oxidative stress may be one of the underlying causes [78]. So far, the conducted research has demonstrated many strategies to combat FASD by either preventing or reducing oxidative stress. This is one of the main mechanisms of many nutrients and drugs associated with the reduction in NADPH level, the increase in a crucial antioxidant—glutathione, the increase in the level of the ADH enzyme, or the reduction in lipid peroxidation. Another important mode of action for both nutritional and pharmacological strategies is the reduction of neuroinflammation and neurotoxicity by reducing levels of pro-inflammatory cytokines. Additionally, it was also observed that certain pharmacological agents may have an impact on epigenetic alterations, changes in synaptic transmission, or dopamine receptor mRNA. The last category of intervention included in the presented review concerns the behavioral strategies. It comprises various activities targeting neuroplastic potential in the brain, sensory processing deficits, deficits in motor performance, changes in the hypothalamic–pituitary–adrenal axis’s responsiveness to stress and deficits in executive functioning. A general summary of the therapeutic interventions effective in reducing serious consequences of PAE that have been studied in animal models is provided in Figure 2.

### 4.1. Nutritional Interventions 

#### 4.1.1. Vitamin E and Beta-Carotene

Studies conducted by Mitchell et al., as early as 1999, in a laboratory setting, presented that supplementation of vitamin E may reduce the harmful effects caused by alcohol in the developing rat hippocampus cultures, even when the alcohol exposure is minimal. Moreover, when supplied at doses of 50 μM, it improved viability (75% with treatment of 2400 mg/dL ethanol and 50 μM Vitamin E compared to 45% with treatment of 2400 mg/dL ethanol alone) and survival rates in the presence of different levels of ethanol exposure, ranging from 16 to 24 g/L. Similar observations were noted when scientists exposed these neuronal cell cultures to ethanol and beta-carotene. In that case, when the hippocampal neuronal cell cultures were treated with both 2000 mg/dL ethanol and 50 μM beta-carotene, the cell viability increased to 55% compared to the control group. This indicates that beta-carotene supplementation also provides potential protection against neurotoxicity in the hippocampal cultures of embryos, induced by alcohol [79,80].

#### 4.1.2. Folic Acid

Folic acid is a vital nutrient that may offer a protective effect against the exposure of ethanol during pregnancy. The research has demonstrated that it functions as an antioxidant, and its mechanism is associated with the reduction of NADPH oxidase in the kidney and liver [78,81,82,83].

In 2001, Cano et al. administered ethanol and folic acid to pregnant rats. The body weight of neonates in the control group, ethanol group, and ethanol plus folic acid treated group increased by 9.06 g, 4.12 g, and 6.56 g, respectively, during a period of 3 weeks. These values were significantly different from each other, suggesting that folic acid may exert protective effects against ethanol. In mechanism-oriented research, the use of folic acid supplementation at a dose of 152 mg per day led to a decrease in oxidative stress and the occurrence of thiobarbituric acid reactive substances (TBARS) in the livers of the group that consumed both ethanol and folic acid [84,85]. Subsequent groups of researchers have analyzed the chemical in the context of organ damage. It has been documented that the administration of folic acid at a dosage of 60 mg/kg effectively counteracted the decrease in fetal brain weight observed in pregnant mice exposed to alcohol [85]. In 2010, Serrano et al. demonstrated that administering folic acid at a dosage of 10.5 mg/kg of the mother’s body weight can effectively prevent the occurrence of cardiac dysfunction in embryonic mice that have been exposed to ethanol prenatally [86]

Similarly, Han et al., in 2012, published the results of research designed to assess the implications of supplementation of folic acid at a dose of 10.5 mg/kg maternal body weight. Their results showed that folic acid is effective in reversing abnormal placenta formation and embryonic development, as well as preventing the restriction of intrauterine growth induced by alcohol in the mice model of pregnancy [87].

Finally, in 2015, Muralidharan et al. utilized a zebrafish model to demonstrate that the supplementation of folic acid (at a concentration of 75 mM) effectively reversed the negative effects of alcohol (at concentrations of 100 mM and 150 mM) on the development of the retina. This reversal was shown in terms of the restoration of normal morphological features, particularly the proper differentiation of the optic nerve and photoreceptor cells, within a period of 2 to 24 h after fertilization [88].

#### 4.1.3. Choline

Choline is a crucial nutrient needed for the proper development of the fetus. Insufficient levels of choline can lead to the occurrence of neural tube abnormalities and malfunction in the central nervous system [89,90]. Alcohol intake during pregnancy results in a deficiency of choline, which leads to neurobehavioral abnormalities. These abnormalities may be caused by variations in neurotransmitter levels, alterations in DNA methylation, and discrepancies in gene expression. These mechanisms are considered to have a crucial role in the molecular cause of FASD [83,91,92].

In 2009, Thomas et al. reported that the administration of choline (250 mg/kg/day) to pregnant rats that were exposed to alcohol (6.0 g/kg/day ethanol via intubation from gestational day (GD) 5–20) resulted in a decrease in the severity of birth and brain weight deficiencies, as well as delays in the emergence of incisors teeth and changes in reflex development [93].

In 2013, Beckdash et al. found that gestational choline supplementation modified the alcohol-induced epigenetic alterations, specifically affecting histone marks in b-EP-producing POMC neurons and DNA methylation in the hypothalamic POMC gene promoter of the adult offspring. Additionally, the choline supplementation normalized the stress response to immunological challenge in rats exposed to alcohol [94].

Another study conducted by Sawant et al. in 2019 supported the hypothesis that choline supplementation (in the dose of 10 mg/kg from GD 4 until the end of pregnancy) can promote fetal growth and alleviate adverse effects in sheep model of binge alcohol drinking corresponding with prenatal alcohol exposure during the first trimester of human pregnancy. Using the ultrasound examination, they showed that choline significantly enhances fetal frontothalamic distance (FTD) and thalamic width (TW) measurements and improves fetal femur and humerus bone lengths [95].

Finally, in 2023, Baker et al. investigated the influence of choline supplementation on the levels of cytokines in the hippocampus of adolescent and adult rats exposed to alcohol during early stages of development. They found that choline decreased age-related alterations in interleukin-1 beta (IL-1β) and interleukin-5 (IL-5), while also reducing the persistent rise in IFN-γ in adults exposed to ethanol. Furthermore, choline had an impact on the level of inflammation by adjusting the proportions of pro- and anti-inflammatory cytokines [96].

#### 4.1.4. Omega-3 Fatty Acids

Omega-3 fatty acids are a type of polyunsaturated fatty acids (PUFAs). Docosahexaenoic acid (DHA) is a type of omega-3 fatty acid, and it possesses antioxidant effects. The optimal neural development of the fetus relies on the mother’s intake and dietary status of DHA, which is abundant in human brains. It has the potential to enhance the movement of receptors, the transfer of signals between neurons, and the flexibility of cell membranes [83].

In 2013, Patten et al. carried out an experiment to assess the influence of n–3 FA (including DHA) supplementation on the concentration of glutathione, an antioxidant that constitutes an essential function in brain and lipid peroxidation in rats. It was found that exposure to ethanol during fetal development led to a decrease in glutathione levels in the brain and an increase in lipid peroxidation, resulting in oxidative stress. Additionally, the group that received n–3 FA supplementation (34.2% n–3 FAs, with 24.6% DHA) experienced an elevation in glutathione levels and a reduction in lipid peroxidation. Therefore, this intervention partially neutralized the adverse effects of PAE [54].

Another study carried out by Wellman et al. in 2015 revealed that rats exposed to ethanol during their prenatal stage exhibited less vocalization, decreased play fighting, and traversed a noticeably smaller gap compared to animals that were not exposed to ethanol. Nevertheless, this research also indicated that the administration of DHA improved the ethanol-induced impairments to such an extent that the mice exposed to ethanol and administered DHA were no longer significantly different from the animals treated as controls [97].

In 2019, Feltham et al. investigated the impact of DHA supplementation during prenatal ethanol exposure on the expression of fetal liver genes associated with oxidative processes. Their experimental model of pregnant Sprague-Dawley dams revealed that exposure to ethanol (EtOH) during the initial 10 days of pregnancy resulted in an increase in the size of the fetal liver. This was accompanied by changes in the gene expression of glutathione reductase (GR) and glutathione peroxidase-1 (GPx1). However, when DHA was included in the maternal diet, these alterations were reversed and returned to normal. This pertains to the increased integration of DHA in phospholipids (PL) and free fatty acids (FFA) in the liver of the fetus [98].

To conclude, these findings suggest that injection of DHA may possess therapeutic potential in counteracting certain detrimental effects caused by ethanol, and including DHA into the diet may serve as a dietary approach to mitigate the oxidative damage caused by alcohol consumption during pregnancy.

#### 4.1.5. Vitamin A

Vitamin A is crucial for the process of cellular differentiation and the proper growth and development of both animals and humans. Maternal vitamin A stores are reduced by alcohol consumption during pregnancy, which can disrupt the proper growth of fetal cells. The suggested explanation of this phenomenon is that when retinol and alcohol are present, the enzyme ADH, which plays an essential part in the rate-limiting step of retinol oxidation, has a greater attraction to alcohol. As a result, it prioritizes the metabolism of alcohol over retinol [83].

In 2010, Marrs et al. conducted a study that revealed that administering zebrafish embryos with a low concentration (10^−9^ M) of retinoic acid (RA) and 100 mM ethanol during the gastrulation and somitogenesis phases effectively alleviated a range of abnormalities caused by treating embryos solely with 100 mM ethanol. RA mitigated such anomalies as early cell movements in gastrulation (anterioreposterior axis), craniofacial cartilage creation, or ear development [99].

#### 4.1.6. Resveratrol 

Due to its anti-inflammatory and anti-oxidant properties, resveratrol is another promising substance that may diminish behavioral deficits resulting from PAE [100]. This is a natural polyphenol phytoalexin found in more than 70 plant species, including red grapes or nuts [101]. In 2011, Tiwari et al. designed a study to examine the impact of resveratrol in cognitive impairments and neuronal apoptosis among rat offspring exposed to ethanol postnatally. The obtained results showed that long-term administration of resveratrol at doses of 10 and 20 mg/kg significantly lowered the behavioral, biochemical, and molecular alterations in various parts of the brain among the ethanol-treated group of offspring. Importantly, this treatment effectively prevented memory deficits, which was achieved by both reducing oxido-nitrosative stress and decreasing the elevated level of pro-inflammatory cytokines and apoptotic markers in various brain regions [100]. Similarly, in 2011, Kumar et al. conducted a study that suggested that resveratrol has neuroprotective effects in the cerebellum by interacting with redox-regulating proteins in an experimental model of FASD. They demonstrated that administration of 100 mg/kg of resveratrol to rat pups on postnatal day 7 effectively inhibited ethanol-induced apoptosis by the elimination of reactive oxygen species in the external granule layer of the cerebellum and promotion of the survival cerebellar granule cells. Moreover, it reversed alterations caused by ethanol in the amount of the transcription factor nuclear factor-erythroid derived 2-like 2 (nfe2I2, often referred to as Nrf2) in the nucleus. As a result, the expression and activity of such gene targets as NADPH quinine oxidoreductase 1 or superoxide dismutase remained unchanged in the cerebellum of pups exposed to ethanol [102].

#### 4.1.7. Summary

Despite the fact that there is limited available research concerning the precise effects of nutrient supplementation in FASD, we conducted a comprehensive evaluation of the reported studies to summarize the most promising substances. The mechanisms of action of these therapeutics primarily involve the reduction of oxidative stress, neuroinflammation, or neurotoxicity. It is also worth mentioning that because of the multiple metabolic impairments, a single nutrient probably is not sufficient to reverse the damage triggered by PAE completely, and a proper strategy should engage a complex therapeutical approach. Finally, the future research should be concentrated not only on the identification of the optimal dosage of nutrients needed to alleviate negative effects of FASD but also on the exploration of the persistent implications of such supplementation.

### 4.2. Pharmacological Interventions

#### 4.2.1. Astaxanthin

In 2014, Zheng et al. conducted a study in which they tested the protective effect of astaxanthin on fetal alcohol spectrum disorder in mice. This is an antioxidant substance with stronger activity than vitamin E. The researchers have observed that embryos of pregnant mice treated with ethanol and astaxanthin did not present any notable difference in the case of growth retardation in comparison to the control group. Moreover, the expression levels of the neural marker genes increased during the process of neural development—Otx1 and Sox2 were considerably reduced to 12.5% and 20% in embryos that received prenatal treatment of 20 mL/kg ethanol, compared to the control group, while in embryos treated with 20 mL/kg ethanol and 100 g/kg/d astaxanthin, the levels of Otx1 and Sox2 expression showed no significant variation. Similarly, astaxanthin improved the decrease in glutathione peroxidase (GPx) levels and the increase in hydrogen peroxide (H_2_O_2_) and malondialdehyde (MDA) levels induced by exposure to ethanol during pregnancy [78,103].

#### 4.2.2. Metformin

Alcohol use during pregnancy leads to an increase in reactive oxygen species (ROS) levels and cell death (apoptosis) in the brain cells of rat fetuses. However, administering antioxidants to the cells beforehand helps prevent cell death [104].

Additionally, glial cells are activated and proinflammatory mediators increase in the brain of rodent models following alcohol use during the period reflecting the third trimester of human pregnancy. This indicates an increased neuroinflammatory response [105].

To find a substance that reduces this negative cellular response, Sabzali et al. conducted a study in which they assessed the beneficial effects of metformin in reducing neuroinflammation caused by ethanol and preventing neuron cell death in the hippocampus of adult male rats in an animal model of FASD. In this study, they administered ethanol in a milk solution with concentrations of 5.25 and 27.8 g/kg, respectively, by intragastric intubation within 2–10 days after birth. The effects were measured using an ELISA method to assess the levels of tumor necrosis factor-α (TNF-α) and antioxidant enzymes. Moreover, immunohistochemical staining was performed to verify the expression of glial fibrillary acidic protein (GFAP) and cleaved caspase-3. As a result, it was revealed that the administration of metformin at a dosage of 40 mg/kg resulted in a notable reduction in the concentration of MDA, while simultaneously elevating the levels of glutathione peroxidase (GSH-Px) and superoxide dismutase (SOD) compared to the ethanol group. Furthermore, it reduced the release of TNF-α caused by ethanol-induced neurotoxicity, diminished the number of cells undergoing cleavage by caspase-3 in the CA1 region of the hippocampus, and lowered the amount of GFAP [106]. To sum up, the potential neuroprotective impact of metformin could be a feasible therapy approach for FASD. Nevertheless, further study is necessary in this area, especially because there is a risk of fetal growth retardation relative to gestational age and a risk of hypertension and nephropathy when metformin is used in pregnancy.

#### 4.2.3. Cannabinoids 

The recent research has demonstrated that some cannabinoids possess neuroprotective properties through several pathways [107]. These chemicals have the potential to be used therapeutically in cases of neuroinflammatory and neurodegenerative illnesses, such as alcohol-induced neuroinflammation [108]. In 2021, García-Baos et al. conducted a study that examined the impact of cannabidiol (CBD) on enduring cognitive impairments caused by early alcohol consumption. They utilized pregnant female mice to simulate alcohol binge drinking during prenatal and lactation periods by providing them with only limited access to a 20% alcohol solution. After exposure to alcohol throughout the prenatal and lactation periods, they administered CBD to both male and female offspring from post-natal day (PD) 25 to 34. Finally, the assessment of their cognitive abilities took place at PD60 [109]. The results of this study indicated that CBD treatment during peri-adolescence improved cognitive abnormalities in FASD-like mouse model, with no variations based on sex. It specifically enhanced reference and object location memories. Similarly, an interaction between variables was discovered for delayed spatial working memory. Additionally, CBD reverses the elevated levels of TNF-α and IL-6 caused by PLAE (prenatal and lactation alcohol exposure) in the hippocampus [109]. Another study, designed by Gasparyan et al. in 2023, aimed to assess the impact of early and chronic CBD treatment on offspring exposed to an animal model of FASD. In this experiment, female mice with a history of alcohol usage were given ethanol through oral gavage at a dose of 3 g/kg every 12 h from gestational day 7 to postnatal day 21. On the weaning day, pups were split by sex and started receiving CBD at a dosage of 30 mg/kg/day intraperitoneally. Behavioral and neurobiological changes were assessed after 4 to 6 weeks [110]. The results were presented that CBD influences the metabolomic alterations observed in the hippocampus and prefrontal cortex. Notably, no alterations were observed in the mitochondria or the oxidative state of the cells. Instead, lipid and protein metabolism may be another pathway responsible for the observed cellular repair following chronic CBD treatment. Modulating the expression of the Pparβ/δ gene could be one of the various targets implicated in CBD’s ability to cause cellular repair and behavioral changes [110]. In conclusion, the presented findings indicate that administering CBD early and repeatedly may influence the enduring behavioral, genetic, and protein changes caused by the FASD model. This suggests CBD seems to be a promising pharmaceutical agent. Nevertheless, further research is needed to assess the varying effects of different dosages of CBD in order to determine the optimal timing and conditions for its administration [109].

#### 4.2.4. Obestatin

In 2019, Toosi et al. carried out an experiment to determine the protective effects of obestatin on alcohol-induced neuronal apoptosis and neuroinflammation in rat pups exposed to postnatal ethanol [111]. Male Wistar rat pups were supplied ethanol by intragastric intubation from postnatal days 2 to 10, which corresponds to the third trimester in humans, and simultaneously, they were administered obestatin at doses of 1 and 5 μg/kg subcutaneously. Thirty-six days post-birth, a spatial memory test was conducted using the Morris water maze test, followed by measuring antioxidant enzymes and TNF-α levels. Glial fibrillary acidic protein (GFAP) and caspase-3 protein expression levels were assessed using immunohistochemical labeling following the behavioral test. The results of these measurements showed a significant reduction in spatial memory deficits, an increase in glutathione and total superoxide dismutase activity, and also, a decrease in the level of malondialdehyde and TNF-α compared to the ethanol group. Moreover, the researchers found a drop in caspase-3 levels and a reduction in GFAP-positive cells in the hippocampus of rat pups exposed to ethanol. The presented conclusions demonstrate the potential role of oxidative-inflammatory-cascade-mediated apoptotic signaling in cognitive impairments caused by postnatal ethanol exposure. It also highlights the neuroprotective effects of obestatin on alcohol-induced behavioral, biochemical, and molecular deficits [111].

#### 4.2.5. Crocin

In 2022, Farhadi et al. assessed the protective effects of crocin on ethanol-induced neuroinflammation and neuronal apoptosis in the hippocampus of rat pups exposed to alcohol during postnatal days [112]. Crocin is the principal substance found in *Crocus sativus* L. (saffron). It has antioxidant properties both in laboratory experiments (in vitro) and in living organisms (in vivo). Crocin inhibits lipid peroxidation, enhances the impact of superoxide dismutase, and increases the synthesis of glutathione. During this experiment, ethanol (5.25 g/kg) was administered in a milk solution (27.8 mL/kg) by intragastric intubation between 2 and 10 days after birth and the animals were administered crocin at doses of 15, 30, and 45 mg/kg between 2 and 10 days after birth. Then, the memory and spatial learning depending on the hippocampus were assessed 36 days after birth with the Morris water maze challenge. Additionally, the TNF-α and antioxidant enzyme concentrations were analyzed using an ELISA assay to check antioxidant and anti-inflammatory properties, and immunohistochemical labeling was used to assess the expression of GFAP, Ionized calcium binding adaptor molecule 1 (Iba-1), and caspase-3. The obtained results indicated that crocin significantly reduced the spatial memory impairment caused by ethanol neurotoxicity. It also significantly increased SOD and GSH-PX levels while decreasing TNF-α and MDA concentrations compared to the ethanol group. Finally, the hippocampus caspase-3 level reduced significantly, and the number of GFAP and Iba-1-positive cells decreased significantly in the crocin group. As the authors stated, the effectiveness of crocin may be linked to alterations in the expression of antioxidant enzymes, endogenous inflammatory mediators, and proteins associated with apoptosis. This leads to a decrease in neuronal death [112].

#### 4.2.6. CE-123

In 2021, Gibula-Tarlowska et al. conducted an experiment to evaluate a novel dopamine transporter (DAT) inhibitor, CE-123, (at doses of 1, 3, or 10 mg/kg) for its ability to mitigate the behavioral abnormalities generated by ethanol in a rat model of FASD. The results of this study presented the changes in the expression of dopamine receptor mRNA (in particular, D1, D2, and D5) in the striatum, hippocampus, and prefrontal cortex of adult rats with PAE. Additionally, CE-123, at doses of 3 and 10 mg/kg, reduced hyperactivity and, at dose of 10 mg/kg, improved the impairment of reversal learning. Therefore, CE-123 could be a beneficial treatment for some deficits linked to neonatal alcohol exposure [113].

Furthermore, observations of the positive impact of the novel atypical dopamine reuptake inhibitor CE-123 on rats neonatally exposed to ethanol were also provided by Socha et al. in 2024. Their study revealed that these male and female rats displayed deficiencies in social novelty discriminating during different stages of adolescence based on their sex and age. The abnormalities were limited to social interactions and were not a result of general deficits in learning and memory. However, the long-term administration of CE-123 prevented the development of these social impairments. CE-123 in the hippocampus of adolescent rats improved BDNF levels and lowered TrkB receptor expression in ethanol-exposed animals, indicating enhanced neuroplasticity during development [114].

Hence, these data suggest that CE-123 could be an effective remedy for some of the impairments linked to PAE.

#### 4.2.7. Pioglitazone

Pioglitazone is a peroxisome proliferator-activated receptor (PPAR)-γ agonist that has been studied in the context of FASD. PPAR-γ, a part of the nuclear receptor protein family, is primarily recognized for its role in regulating glucose and lipid metabolism. Thiazolidinediones, including pioglitazone, are PPAR-c agonists initially identified for their ability to influence these metabolic processes. These compounds are frequently utilized in treating type II diabetes, owing to their metabolic regulatory properties [115]. In recent studies, PPAR-γ agonists have been shown to have anti-inflammatory properties, including in the central nervous system [116].

Kane et al., in their third trimester-equivalent FASD model, treated mice with PPAR-γ agonists before the administration of alcohol. They found that pioglitazone administration protects Purkinje cell neurons, as well as microglia in the cerebellum. The research indicates that agonists of PPAR-γ could potentially play a significant role in reducing the harmful effects of ethanol on the developing central nervous system [117].

The later study from Drew and colleagues focuses on ethanol-induced neuroinflammation with expression of pro-inflammatory molecules and microglial activation. They report that pioglitazone administration before ethanol exposure blocks microglial activation and production of the cytokines and chemokines in the hippocampus, cerebellum, and cerebral cortex of neonatal mice. However, further studies need to determine whether pioglitazone treatment is effective at different times during ethanol exposure and if the effects of PPAR-γ agonist administration are long-lasting [118].

#### 4.2.8. Apelin-13

Neuroinflammation and ROS production are mechanisms that have a significant meaning in terms of neurodegeneration that we can observe in FASD individuals [119]. That observation led to a hypothesis that anti-inflammatory agents may be effective in the treatment of FASD; one such agent is apelin. Apelin-13, a major apelin isoform in the central nervous system and cardiovascular system, is a 13-amino acid peptide that serves as the endogenous ligand for the apelin receptor, also known as the APJ receptor. It exhibits various biological activities, including hypotensive, neuroprotective, and cytoprotective effects. Apelin-13 has a multisystemic protective effect and is involved in the regulation of various pathophysiological mechanisms such as apoptosis, neuroinflammation, angiogenesis, and oxidative stress. It is currently the subject of extensive research involving the nervous system [120].

Mohseni and colleagues evaluated the protective effect of apelin-13 on the third-trimester equivalent rat model of FASD. Apelin was administered subcutaneously immediately after ethanol exposure. The findings indicate that apelin-13 mitigates impairments associated with ethanol exposure in the water maze task, which relies on hippocampal functions related to memory and learning. These deficits are possibly ameliorated due to the anti-inflammatory properties of apelin. It increased the expression of antioxidant enzymes, decreased the level of apoptotic-associated proteins, and attenuated TNF-α production, as well as lipid peroxidation, resulting in a decrease in neuronal cell death [121]. The research shows apelin-13 as a promising therapeutic choice for FASD; however, further studies are needed to assess the effects of apelin on other brain regions and deficits that accompany them.

#### 4.2.9. Epigallocatechin Gallate

Epigallocatechin gallate (EGCG) is a natural compound found in green tea, which has been linked to numerous health benefits. It is a type of catechin, which is a plant compound with antioxidant properties. EGCG has been studied for its potential effects on human health, including its antioxidant, anti-inflammatory, and anticancer properties [122].

EGCG probably reduces the risk of cognitive impairment in children with FASD. Tiwari et al. in their research show that the administration of EGCG in the third-trimester equivalent rat model of FASD ameliorated behavioral impairments resulting from postnatal ethanol exposure by modulating oxide-nitrosative stress and reducing elevated levels of pro-inflammatory cytokines (TNF-α and IL-1β), NF-κB, and caspase-3 across various cerebral regions in ethanol-exposed offspring [123].

In a mice model of FASD, Long et al. found that EGCG can prevent some of the embryonic injuries caused by ethanol. Pregnant female mice were given EGCG on gestational day 7 (G7) and G8, along with ethanol on G8. The oral administration of EGCG shows antioxidative properties, and by significantly reducing ethanol-induced hydrogen peroxide (H_2_O_2_) and malondialdehyde (MDA) production, it protects murine embryos against developmental delays induced by ethanol exposure [124].

In conclusion, the research conducted by Almeida-Toledano et al. underscores the potential of EGCG as an effective antioxidant treatment for mitigating the effects of prenatal alcohol exposure. Following the previous reports, their findings on the mouse model of FASD demonstrate that EGCG reduces the oxidative stress generated by ethanol, thereby reducing its teratogenic impact. Moreover, they studied the influence of EGCG on abnormal placental development caused by ethanol exposure. They report that EGCG diminishes the alcohol-induced alternations in placental angiogenic factors, promoting healthier placental function and fetal development [125].

The studies on rodent models of FASD show that EGCG is effective in ameliorating the effects of prenatal ethanol exposure during the equivalent of all three trimesters of human development. Subsequent research should concentrate on assessing the impact of EGCG therapy in neonatal human equivalent, given that initiating treatment during gestation may not always be feasible.

#### 4.2.10. Curcumin

Turmeric is a spice derived from the root of the Curcuma longa plant, which is native to Southeast Asia and the Indian subcontinent. It is commonly used in traditional medicine and cooking, particularly in Indian cuisine. Turmeric contains several bioactive curcuminoids, with curcumin being the most well-known and studied. Curcumin is a polyphenol compound found in turmeric and is responsible for its yellow color and many of its health benefits. Curcumin has been studied for its various potential health benefits, including anti-inflammatory, anticarcinogenic, anti-ageing, and antioxidant properties [126,127].

Studies utilizing the zebrafish model for FASD demonstrated that turmeric supplementation mitigated the retardation of body length associated with ethanol exposure. Embryos exposed to ethanol and co-treated with high doses of curcuminoids from turmeric extract exhibited body lengths comparable to those of the control group. Meanwhile, embryos receiving lower doses of curcuminoids presented increased body lengths relative to the ethanol-only group, though these did not match the control group’s lengths [128].

Cantacorps et al.’s research examined the impact of curcumin treatment on correcting behavioral impairments and molecular changes due to prenatal ethanol exposure in C57BL/6 mice. Prenatally exposed male mice received curcumin injections daily from postnatal days 28 to 35. The findings suggest curcumin enhances cognitive abilities in adult mice, improving working and recognition memory. Curcumin exhibited anti-inflammatory effects by reducing IL-6 and NF-κB expression in the hippocampus and prefrontal cortex, and it also lowered Iba-1 and GFAP levels, indicating reduced microglial activation and astrogliosis. Nonetheless, curcumin did not affect the epigenetic alterations induced by prenatal ethanol exposure, and it did not change the increased activity of histone acetyltransferase [129].

#### 4.2.11. Trichostatin A

Trichostatin A (TSA) is a substance commonly used in the scientific research to investigate gene expression and epigenetic modifications. As a histone deacetylase inhibitor (HDAC inhibitor), TSA works by inhibiting histone deacetylase, leading to increased acetylation of histone proteins and a more open chromatin structure that promotes active transcription. This can have an impact on the expression of genes involved in numerous cellular processes, including cell cycle regulation, differentiation, and apoptosis. In the case of FASD, TSA has been found to block the activity of HDACs induced by prenatal ethanol exposure. This inhibition promotes the active transcription of neuronal genes, ultimately aiding in the improvement of plasticity and cognitive deficits.

The study conducted by Montagud-Romero et al. reveals that TSA can improve most of the behavioral changes observed in C57BL/6 mice prenatally exposed to alcohol. The male offspring exposed to ethanol and treated with TSA during postnatal days 28–35 showed enhanced working memory compared to the group only exposed to alcohol. Additionally, TSA reversed the reduction of neurogenesis in the dentate gyrus, which is induced by alcohol. The research underscores the role of HDACs in the cognitive and emotional impairments associated with FASD and suggests that HDAC inhibitors, such as TSA, may hold potential as a treatment option [130].

Shivakumar et al. investigated the neuroprotective effects of Trichostatin A (TSA) therapy against postnatal ethanol exposure (PEE) in mice, revealing TSA’s capacity to mitigate neurodegeneration and synaptic plasticity impairments. TSA treatment before PEE preserved histone acetylation levels, preventing caspase-3 activation and the loss of critical synaptic plasticity-related genes, Egr1 and Arc. Similarly to Montagud-Romero, they found that treatment with TSA ameliorates alcohol-induced memory, learning, and social interaction deficits. These outcomes highlight TSA’s potential in reversing the epigenetic and neurobehavioral deficits induced by PEE, suggesting its therapeutic viability for addressing FASD-related cognitive declines through epigenetic remodeling in the brain [131]. It is worth mentioning that trichostatin A (TSA), as a pan-HDAC inhibitor, affects various cell types, not limited to those in the central nervous system. Therefore, it is imperative to delineate its impacts across different bodily regions before considering TSA for therapeutic applications in humans.

#### 4.2.12. Varenicline

Varenicline is a medication that acts as an agonist of the nicotinic receptors. It is primarily used in the treatment of smoking cessation. Due to its cholinomimetic properties, it can have similar positive effects as other cholinergic medications that have been tested in the treatment of FASD. In addition to its ability to alleviate some of the deficits caused by ethanol, varenicline also reduces the consumption of alcohol, which could be beneficial in preventing maternal alcohol consumption [132].

A study was conducted by Montgomery and colleagues to assess the effectiveness of varenicline in treating behavioral deficits observed in FASD. The study utilized pregnant rats exposed to ethanol during the equivalent of the third trimester of human pregnancy. The results indicated that rats treated with both varenicline and ethanol exhibited fewer learning deficits and reduced anxiety compared to those exposed to ethanol alone. Moreover, the team examined the physiology of the basal forebrain (BF) and found that varenicline treatment led to a decrease in ethanol-induced changes in synaptic transmission. Since early neuronal development relies on nicotinic signaling, alterations in BF signaling may account for the behavioral dysfunction seen in individuals with FASD. The researchers also recognized the sexual dysmorphism of ethanol-induced deficits and called for further research on male rat models of FASD [133].

Bariselli et al. reported that sex-specific anatomical and motor deficits were observed in a prenatal ethanol exposure mice model that mimics the third-trimester equivalent. In their study, they found that impaired motor skills were associated with increased striatal dopamine release and impaired nicotinic regulation of electrically evoked dopamine release. The study also suggests that the dysfunction of nicotinic acetylcholine receptors (nAchR) may contribute to the observed behavioral deficits seen in individuals exposed to alcohol. The administration of Varenicline in ethanol-exposed female mice helped to improve motor skills deficits in rotarod training. Based on these results, it is suggested that nAChR agonists could be a potential treatment for FASD [134].

#### 4.2.13. Nicotinamide

Nicotinamide is an amide form of niacin (vitamin B3) that plays a crucial role in energy production and DNA repair. It is a precursor for the coenzyme β-nicotinamide adenine dinucleotide (NAD+), playing a crucial role in generating ATP within the mitochondrial electron transport chain. Recently, it has emerged as a promising cytoprotectant for the brain, particularly in the context of acute and chronic neurodegenerative disorders. It operates by maintaining DNA integrity and preserving membrane asymmetry to prevent inflammation, cellular phagocytosis, and vascular thrombosis. Its protective mechanisms involve the modulation of various cellular and molecular pathways, including Akt activation, preservation of mitochondrial membrane potential, and regulation of caspase activities, independent of intracellular pH and mitogen-activated protein kinases [135].

In a study by Ieraci et al., nicotinamide was investigated as a potential treatment for the neurodegenerative effects of ethanol. The study was conducted on the third-trimester equivalent of the FASD mouse model. Nicotinamide was administered between 0 to 8 h after ethanol exposure on postnatal day 7. The study results showed that the administration of nicotinamide inhibited caspase-3 activation and subsequent neuronal cell death in the hippocampus, thalamus, and cingulate cortex. The treatment’s behavioral effects were also assessed, and the results showed that nicotinamide administration prevented ethanol-induced memory impairment in contextual fear conditioning and reduced hyperactivity in the open field and the plus maze tests [136].

A recent study conducted by Ieraci and colleagues explored the impact of prenatal alcohol exposure on a different brain region. The team utilized a mouse model akin to the one employed in a previous study, administering nicotinamide to the mice immediately or two hours following exposure to ethanol on postnatal day 4. The study demonstrated that nicotinamide treatment was able to decrease neurodegeneration caused by ethanol in the cerebellum. Specifically, nicotinamide inhibited the caspase-3 activation and poly (ADP-ribose) polymerase (PARP-1) over-activation, both of which are mechanisms of ethanol neurotoxicity. These results imply that nicotinamide administration following ethanol exposure may have the potential to treat FASD and other neurodegenerative conditions. However, further research is necessary to validate these findings [137].

#### 4.2.14. Neuroprotective Peptides

Synthetic peptides, known as SALLRSIPA (SAL or ADNF-9) and NAPVISIPQ (NAP), are derived from proteins such as activity-dependent neurotrophic factor (ADNF) and activity-dependent neuroprotective protein (ADNP), which are expressed during brain development. These peptides are synthesized and released by astroglia in response to vasoactive intestinal peptide (VIP) stimulation. The research has shown that SAL and NAP possess neuroprotective properties and can mitigate some of the harmful effects associated with prenatal exposure to ethanol on the developing brain [138].

According to research conducted by Incerti et al., administering NAP and SAL can improve certain behavioral deficiencies observed in mice exposed to ethanol during gestation. Specifically, NAP and SAL were administered postnatally to prenatally alcohol-exposed mice for ten days. The peptides were successful in reversing learning deficits, as evidenced by T-maze and Morris water maze learning tests. It is believed that the restoration of N-methyl-D-aspartate receptors in the hippocampus and cerebral cortex is responsible for these results. It was found that NAP and SAL treatment resulted in the downregulation of NR2A and NR2B in the hippocampus, while NR2A was upregulated in the cortex, and NR2B was upregulated in the cortex and cerebellum [139]. 

There are a few pathways in which NAP and SAL show their protective properties. One of them is the γ-aminobutyric acid A (GABA) receptor subunit GABAβ3 expression, which plays a critical role in the nervous system. The NAP plus SAL treatment prevented the decrease in GABAβ3 expression 10 days after ethanol exposure [140]. Neuroprotective effects of NAP and SAL might be also mediated by the expression of brain-derived neurotrophic factor (BDNF). In an FAS mice model, pretreatment with NAP plus SAL prevented the alcohol-induced rise in BDNF expression at 24 h and 10 days after alcohol exposure [141].

Roberson et al. conducted a study on an FAS mice model to investigate the role of cytokines in the mechanism of the prevention of alcohol-caused neurodevelopmental anomalies by NAP plus SAL therapy. They found that mice treated with alcohol and peptides did not exhibit an increase in the levels of interleukin (IL)-6, keratinocyte chemoattractant cytokine (KC), and granulocyte colony-stimulating factor (G-CSF). The prevention of the increase in these cytokines, which are known to affect NMDA receptors, may be one of the pathways by which NAP and SAL prevent alcohol-induced pathologies [142].

Sari and their team conducted research to study the potential neuroprotective effects of SAL and NAP peptides, as well as their mechanism of action. Using an FASD mice model, they discovered that prenatal treatment with SAL prevented alcohol-induced fetal brain weight reduction and cell death. Additionally, their analysis of the fetal brain revealed that SAL upregulated several crucial proteins involved in cell division, growth, and transcriptional regulation [143].

In a subsequent study, the team found that ADNF-9 also protected against brain weight loss and cell death in an FASD mice model. ADNF-9 was shown to prevent alcohol-induced increases in phospho-c-Jun N-terminal kinase (JNK) and downregulation of the survival factor Bcl2 family. These major mitochondrial signaling pathways may be contributing factors to the neuroprotective effect [144]. Furthermore, the combination of SAL and NAP produced similar neuroprotective results [145].

#### 4.2.15. Summary

Given that the adverse effects of PAE appear to result from the elevation of oxidative stress, neuroinflammation, and/or neurotoxicity, medications that reduce these processes may be beneficial in alleviating symptoms of FASD. A multitude of experimental studies have been conducted by scientists to evaluate the effects of substances on the functioning of offspring with FASD. Nevertheless, it appears that a single breakthrough cure remains elusive. In some cases, the substances were administered simultaneously with ethanol to assess their effectiveness in minimizing the detrimental consequences. This co-administration was applied in the case of astaxanthin or metformin to verify the impact of these substances on the reduction of oxidative stress. Another model of treatment implemented in the presented experiments concerns the studies in which pharmacological intervention was administered after PAE, such as apelin-13 or trichostatin. These experiments yielded insights into the potential for reversing long-term complications, including improvements in memory and learning, due to anti-inflammatory properties. Additionally, one substance, pioglitazone, was administered before PAE, which was demonstrated to block microglial activation and the production of cytokines and chemokines in the hippocampus. However, further studies are necessary to confirm the efficacy of pioglitazone treatment at different times of ethanol exposure and to determine the long-term effects of this treatment. 

In conclusion, although the reports are promising, further research is required in this area to gather more evidence of both the efficacy and safety of the proposed treatments and to determine the optimal time to use them.

### 4.3. Behavioral Interventions

Behavioral interventions in animal models of FASD have been studied to mitigate the negative impacts of prenatal alcohol exposure. Some of the interventions studied include aerobic exercise, environmental complexity, and rehabilitative motor training. These interventions hold promise for the development of therapeutic strategies for individuals affected by FASD.

#### 4.3.1. Aerobic Exercise

An aerobic exercise intervention used in the FASD animal model is wheel running (WR). Studies have demonstrated that WR can have a positive impact on various measures of neuroplastic potential in rodents, including adult neurogenesis rates, angiogenesis, and expression of neurotrophic factors. This intervention has also been shown to mitigate behavioral and neuroanatomical aspects of the negative impacts of teratogens (i.e., developmental alcohol exposure) and age-related neurodegeneration in rodents [146,147].

The hippocampus is a key area of interest in understanding the effects of voluntary exercise on cognitive function in the FASD animal model. Several studies have shown that hippocampal structure can be compromised in FASD animal models [148,149]. The hippocampus plays a crucial role in cognitive function, including learning, and spatial navigation [150]. Most studies suggest that adolescent rats who have free access to running wheels and engage in voluntary exercise show an increase in hippocampal and cortical neuroplasticity [147,151,152,153].

Wheel running and environmental complexity can be coupled together to form, as named by the author, a “superintervention”. These two interventions combined act in a synergistic way with environmental complexity enhancing the short-term beneficial results of wheel running. Wheel running and environmental complexity have been shown to increase hippocampal neurogenesis and reduce some of the behavioral and neuroanatomical aspects of developmental exposure to alcohol and age-related neurodegeneration in rodents [146,147]. However, it is difficult to determine the relative contributions of each component of the intervention, and further research may address the optimal ratio of wheel running access to environmental complexity access.

It is important to not only verify the positive results of voluntary exercise at the morphological level, but also, it is crucial to assess functional deficits in the FASD animal model. One of the possible methods to measure functional deficits in prenatally exposed animals is trace conditioning. Trace fear conditioning is designed to measure hippocampal function. It involves a neutral conditioned stimulus, such as a flash of light, and an aversive unconditioned stimulus, like a shock, separated in time by a trace interval. The trace interval between the flash and the shock critically involves the hippocampus and can be used to evaluate hippocampal-dependent learning and memory [154].

Positive neuroanatomical changes to the hippocampus after intervention do not always correlate with the functional deficits seen in trace conditioning. The results reported by Schreiber et al. show that voluntary exercise and environmental complexity do not reverse deficits in trace and context conditioning that occur after ethanol exposure. However, they did increase context conditioning, another similar form of hippocampus-dependent learning [147]. In contrast to those results, a follow-up study showed that combined wheel running and environmental complexity intervention did reverse the deficits in conditioned fear to the levels of the control group [152].

In functional deficits reported by Schreiber et al., the additional role is played by the prefrontal cortex which receives projections from hippocampal formation. The medial prefrontal cortex (mPFC) also participates in the long-term storage of fear memory [155]. Hamilton et al. used an animal model of binge drinking during the third trimester of pregnancy and found that voluntary exercise partially mitigated alcohol-induced deficits in the dendritic complexity of the mPFC neurons in male adolescent rats. The study observed that alcohol exposure during the neonatal period stunted developmental growth, but voluntary exercise showed potential beneficial effects in reversing the alcohol-induced damage to the dendritic morphology of mPFC neurons [151].

The influence of aerobic exercise on the corpus callosum, another area affected by prenatal alcohol exposure, is a subject of recent studies. PAE results in impaired executive function and perceptual learning in individuals with FASD; these deficits are associated with a disruption of growth and myelination of the corpus callosum. Adolescent exercise, a stimulator of melanogenesis, was studied to alter these changes. In a neuroimaging study by Milbocker et al., voluntary exercise in alcohol-exposed rats did not alter the corpus callosum myelination process; however, it improved corpus callosum growth in both control and alcohol-exposed rats [156]. Whereas in the follow-up histological examination study, the number of mature oligodendrocytes (OPCs) in the female brain increased after voluntary exercise, which could help upregulate CC myelination and improve interhemispheric communication and cognitive function in individuals affected by FASD [157]. Both of these studies indicate that there are sexual dimorphisms in the effect of voluntary exercise on CC myelination. As a result of this, it is important to determine in future preclinical trials the most effective duration and introduction time for the intervention. In addition to the corpus callosum, exercise has a positive effect on microglial development. Microglia play a crucial role in maintaining synaptic form and function. Voluntary exercise in the form of running alters microglial density and cerebellar volume in a way opposite to the effect of developmental alcohol exposure. The functional implications of these changes need further investigation; however, the results of this study are another promising report for aerobic exercise to improve developmental alcohol exposure deficits [158].

#### 4.3.2. Environment Enrichment

Environmental enrichment (EE) has been studied as a therapeutic intervention in animal models of various conditions, including FASD. EE involves exposing individuals to new and enriched environments that offer opportunities for social, motor, cognitive, and sensory stimulation. Studies on animals have shown that providing an enriched environment can improve behavioral performance and reduce some of the deficits caused by PAE [146,147,152,159].

Wang et al. focused their work on processing the deficits in sensory modalities seen in individuals with FASD. Using a second-trimester-equivalent binge-drinking prenatal ethanol exposure rat model, they found that PEE impairs sensory processing and habituation to visual stimuli, which are normalized by the enrichment of postnatal environmental conditions. The results of the study were interpreted based on the rats’ responses to the light reinforcement experiment. The proportion of active responses was used to assess the effectiveness of the contingent light-onset as a reinforcer. PAE rats responded more to the contingent light onset than control rats in the standard housing condition, but no differences were found between control and PAE rats in the enriched condition. The results also showed that enriched conditions led to a reduction in responding to the designated inactive hole in both control and PAE rats. The impaired habituation observed in PAE rats is consistent with sensory processing deficits. The study suggests that postnatal environmental enrichment can normalize the effects of prenatal ethanol exposure on sensory processing and habituation of visual stimuli in rats [160].

Individuals prenatally exposed to ethanol may have an increased propensity for later self-administration of ethanol or other substances [161,162]. Wang and colleagues verified if environmental enrichment including neonatal handling and post-weaning complex housing would ameliorate this predisposition to drug addiction resulting from prenatal alcohol addiction. The study revealed significant interaction effects between prenatal treatment and rearing condition, as well as between dose and prenatal treatment, indicating the impact of environmental factors on the risk of addiction. Additionally, the main effects of the session and litter were observed, highlighting the complexity of the factors influencing addiction risk. The findings suggest that environmental enrichment may have a protective effect against the development of addiction in individuals with a history of prenatal ethanol exposure [163].

The later study of Aghaie et al. focuses on morphological changes that may contribute to an increased risk of drug addiction and the influence of EE on those changes. Alcohol exposure causes dysfunction of the midbrain dopaminergic neurons, especially those located in the ventral tegmental area, which might account for attention deficits and increased addiction risk [164]. The cause of alcohol-mediated changes in dopaminergic neurons is not certain, although ethanol dependent microglia activation can cause neuroinflammation and ameliorate synaptic maturation during development. The environmental complexity in the second-trimester-equivalent model did not reverse PE changes in dopaminergic midbrain neurons. However, it did reverse the reduction in microglial branch and junction numbers in PE animals, which is a state of microglial activation [165].

#### 4.3.3. Rehabilitative Motor Training

Rehabilitative motor training using complex motor learning has been used in animal models of FASD to rescue deficits in motor performance. This training involves complex motor tasks like negotiating obstacle courses, which include climbing, traversing, and balancing activities. Tasks are designed to improve motor skills, coordination, and neural development, ensuring the engagement of cerebellar motor circuitry that may have been adversely affected by PAE. The intervention shows significant effects on motor coordination and balance, particularly in the cerebellum, and can ameliorate motor and learning deficits resulting from neonatal alcohol exposure. The studies demonstrate that the cerebellum retains plasticity and can respond to rehabilitative training even in adulthood, suggesting the potential for postnatal environmental interventions to mitigate FASD effects [159].

Mastering intricate motor skills can mitigate the impact of PAE on both motor and learning abilities, particularly in the cerebellum. However, the motor tasks used to evaluate the effectiveness of this intervention closely resemble the rehabilitative training activities themselves. Wagner et al. verified a hypothesis that rehabilitative training involving 20 days of training on traversal of an obstacle course would ameliorate the deficits in classical conditioning of eyeblink responses produced by neonatal alcohol exposure. Eyeblink classical conditioning is a cerebellar-dependent learning task but is very different from rehabilitation training itself. During the study, they observed that rehabilitation training effectively reduced alcohol-induced deficits in female acquisition of eyeblink conditioning but not in males. The results of the study show that rehabilitative motor training effectively reverses PAE effects in a cerebral-dependent learning task that is completely different from motor activities learned in training. Presumably, the effects of this intervention reach further than the paramedian lobule, which was indicated in the previous studies [166], and also induce neuronal plasticity in other regions of the cerebellum. The researchers emphasize the necessity of acknowledging gender differences in the results when applying these findings to human contexts due to the varying outcomes observed between male and female subjects. Furthermore, the future research should precisely delineate the nature and form of rehabilitative motor tasks in humans, ensuring they are optimally designed to replicate the effects observed on the cerebellum in animal studies [167].

#### 4.3.4. Neonatal Handling

Neonatal handling is a procedure that involves briefly separating newborn animals from their mother and/or littermates and “gentling” them during the separation. This procedure has been shown to improve post-weaning learning, facilitate maturation, and modify neuroendocrine responses to stressful stimuli in normal animals. However, the effectiveness of the procedure depends on various factors, including species, strain, and sex of the animals, timing variables, housing conditions, maternal behavior, and the neurobehavioral outcome assessed. The effects of neonatal handling are largely mediated by changes in the hypothalamic–pituitary–adrenal axis’s responsiveness to stress. While neonatal handling has been used as a potential treatment for FASD in animal models, it only modifies some but not all effects of prenatal exposure. The effects of neonatal handling on behavior, physiology, and neural function across the lifespan are nuanced, and both beneficial and negative outcomes may occur, depending on the parameters of testing, sex of the subject, and the neurobehavioral system analyzed [168]. Neonatal handling as a potential intervention for ameliorating the effects of prenatal ethanol exposure in rats has been thoroughly revised in the work of Hannigan and colleagues [159].

#### 4.3.5. Working Memory Training

Working memory training is another intervention that could be effective in ameliorating some of the deficits in executive functioning, especially in combination with choline supplementation. The results of the work by Waddell et al. suggest that working memory training, along with choline supplementation, may have a positive impact on cognitive deficits in alcohol-exposed rats. The study used an attentional set-shifting task to assess cognitive flexibility and found that a combination of these interventions showed a synergistic effect in improving the cognitive deficits caused by prenatal exposure to ethanol. Resting-state functional magnetic resonance imaging showed that the functional connectivity among brain regions was different between the sexes and was altered by PEE and by choline plus training. Together, these findings indicate that prenatal exposure to low doses of ethanol has persistent effects on brain functional connectivity and behavior, that these effects are sex-dependent, and that an adolescent intervention could mitigate some of the effects of prenatal ethanol exposure [169]. The present findings demonstrate that prenatal exposure to low levels of ethanol has persistent effects on brain functional connectivity and behavior, with a significant variation between genders. Moreover, the findings suggest that an intervention during adolescence can effectively mitigate some of the adverse effects of PEE.

#### 4.3.6. Summary

It is essential to recognize that the effects observed in animal models may not directly correspond to those in humans. When considering behavioral interventions in the FASD animal model, it is crucial to account for the fact that sex-, postnatal age-, and species-specific differences are critical factors in how specific environments may influence brain development. These differences highlight the importance of carefully interpreting and translating findings from animal research to human contexts, particularly when developing and implementing behavioral interventions for FASD. While animal models provide valuable insights, it is important to exercise caution and consider the limitations when applying these findings to human populations. Aerobic exercise and environmental enrichment especially coupled together give the most promising result and potentially they can ameliorate some of the behavioral and cognitive deficits seen in individuals with FASD.

## 5. Conclusions

Animal studies have provided valuable insights into the pathophysiology of FASD, forming the basis for implementing potential therapies with increasingly precise mechanisms. Among a number of advantages and disadvantages, the choice of an appropriate animal model depends on the specifics of a particular study and requires a decision on the dominant focus of our intervention analysis. It depends on the biological level of organization that interests us most, as well as the need to analyze both structural and functional changes that best reflect those observed in humans. Based on the knowledge of the pathophysiology of FASD development, yet limited, the reports in recent years indicate that there is considerable potential for treating the disease. On one hand, preventive interventions are studied to reduce the severity of alcohol’s toxic effects during the prenatal stage. This includes compounds that are essential for normal fetal development and whose deficiency is caused by alcohol, such as folic acid, choline, vitamin A, or vitamin E, as well as commonly used pharmacological antioxidants, including hypoglycemic agents like metformin or thiazolidinediones. However, at this stage, data on several newly investigated compounds that decrease pro-inflammatory cytokine concentrations and impact the expression of oxidative stress and/or neurodevelopment-related processes appear to be only a starting point for further research. On the other hand, therapeutic interventions can be used to reduce the severity of long-term complications during the postnatal period addressing the behavioral and neuroanatomical deficits associated with FASD. Some of the novel compounds recently tested, particularly synthetic neuropeptides and dopamine transporter inhibitor (CE-123), have shown the potential to reduce deficits in memory, learning, or social behavior. Furthermore, behavioral techniques, such as aerobic exercise, that modulate functional deficits induced by PAE are gaining support in experimental studies due to their effects on morphological changes, such as microglia structure and myelination processes. While this research is promising, the need for early diagnosis and treatments that build on individual strengths is critical to optimizing health outcomes for FASD patients, and the data from animal studies may be helpful in this process.

## Figures and Tables

**Table 1:** Comparison of animal models used to study the effects of alcohol.

Animal Model	Advantages	Disadvantages
Mouse	Ease of care	Occurrence of third trimester equivalent after birth
	Genetic and physiological similarities to humans	
	Extensively described teratogenic effects	
Rat	Larger than mice	
	More sophisticated behavior	
Zebrafish	Ease of incorporation changes in model	Lack of maternal factor affecting metabolism of alcohol
	Rapid embryonic development	
	Possibility of assessing behavioral outcomes	
Xenopus	Relatively large size of embryo	
	Ease of incorporation changes in model	
Avian embryo	Cost-effective and attainable	
	Simplicity in maintenance	
*C. elegans*	Rapid development	
	Fully mapped and sequenced genome	Inability to assess simple behaviors
	Small and well-characterized nervous system	Hard to measure alcohol concentration in embryos
Drosophila	Rapid development	Impermeable to alcohol eggshell
	Simple genome	Inability to assess cognitive impairment
	Cost-effective	
